# Short term safety of magnetic sphincter augmentation vs minimally invasive fundoplication: an ACS-NSQIP analysis

**DOI:** 10.1007/s00464-024-10672-7

**Published:** 2024-02-09

**Authors:** Paul Wisniowski, Luke R. Putnam, Shea Gallagher, Rushil Rawal, Caitlin Houghton, John C. Lipham

**Affiliations:** 1 Division of Upper GI and General Surgery, Department of Surgery, Keck Medical Center of University of Southern California, 1510 San Pablo Street, HCC I, Suite 514, Los Angeles, CA 90033 USA; 2 California University of Science and Medicine, Colton, CA USA

**Keywords:** Magnetic sphincter augmentation, Fundoplication, MSA, GERD, Gastroesophageal reflux disease

## Abstract

**Purpose:**

Magnetic Sphincter Augmentation (MSA) is an FDA-approved anti-reflux procedure with comparable outcomes to fundoplication. However, most data regarding its use are limited to single or small multicenter studies which may limit the generalizability of its efficacy. The purpose of this study is to evaluate the outcomes of patients undergoing MSA vs fundoplication in a national database.

**Materials and Methods:**

The 2017–2020 American College of Surgeons National Surgical Quality Improvement Program (ACS-NSQIP) Registry was utilized to evaluate patients undergoing MSA or fundoplication. Patients requiring Collis gastroplasty, paraesophageal hernia repair, and emergency cases, were excluded. Patient outcomes included overall complication rates, readmissions, reoperations, and mortality.

**Results:**

A total of 7,882 patients underwent MSA (*n* = 597) or fundoplication (*n* = 7285). MSA patients were younger (51 vs 57, *p* < 0.001), and more often male (49.6 vs 34.3%, *p* < 0.001). While patients undergoing MSA experienced similar rates of reoperation (1.0 vs 2.0%, *p* = 0.095), they experienced fewer readmissions (2.2 vs 4.7%, *p* = 0.005), complications (0.6 vs 4.0%, *p* < 0.001), shorter mean (SD) hospital length of stay(days) (0.4 ± 4.3 vs 1.8 ± 4.6, *p* < 0.001) and operative time(min) (80.8 ± 36.1 vs 118.7 ± 63.7, *p* < 0.001). Mortality was similar between groups (0 vs 0.3%, *p* = 0.175). On multivariable analysis, MSA was independently associated with reduced postoperative complications (OR 0.23, CI 0.08 to 0.61, *p* = 0.002), readmissions (OR 0.53, CI 0.30 to 0.94, *p* = 0.02), operative time (RC − 36.56, CI − 41.62 to − 31.49. *p* < 0.001) and length of stay (RC − 1.22, CI − 1.61 to − 0.84 *p* < 0.001).

**Conclusion:**

In this national database study, compared to fundoplication MSA was associated with reduced postoperative complications, fewer readmissions, and shorter operative time and hospital length of stay. While randomized trials are lacking between MSA and fundoplication, both institutional and national database studies continue to support the use of MSA as a safe anti-reflux operation.

**Supplementary Information:**

The online version contains supplementary material available at 10.1007/s00464-024-10672-7.

Gastroesophageal disease (GERD) is one of the most common chronic gastrointestinal disorders with an estimated national prevalence of 20% [1]. The pathophysiology of GERD is multifactorial, but there is consensus that the disruption or displacement of the lower esophageal sphincter results in retrograde flow of gastric contents into the esophagus [2]. Patients’ quality of life if often affected by GERD and its associated regurgitation, heartburn, cough, chest pain, dysphagia, and asthma, one or more of which may be present in up to 60% of patients [3].

The prevalence of GERD is likely underreported due to frequent self-medication via over-the-counter medications [4]. Nevertheless, initial management relies on lifestyle modifications and pharmacological treatment with acid suppression medications [4]. While satisfaction rates on medical management are reported to be around 50%, patients with refractory symptoms, medical noncompliance, presence of hiatal hernia, or those who do not desire to take lifelong medications may be referred for surgical therapy [5].

The most common surgical approach to GERD is a fundoplication. However, long term side effects such as gas and bloating are reported to be around 30% [6]. An alternative surgical modality for GERD is magnetic sphincter augmentation (MSA), which has gained adoption over the past 15 years. Short- and long-term studies have demonstrated equivalent efficacy between fundoplication and MSA in GERD outcomes while maintaining a limited side effect profile. This purpose of this study is to evaluate nationwide outcomes of MSA compared to fundoplication.

## Material and methods

### Data source

The American College of Surgeons National Surgical Quality Improvement Database (NSQIP) is comprised of data from over 600 participating hospitals. Data from NSQIP 2017–2020 was included in this study. Institutional Review Board approval was not required for this study.

### Study population

**Fig. 1:**
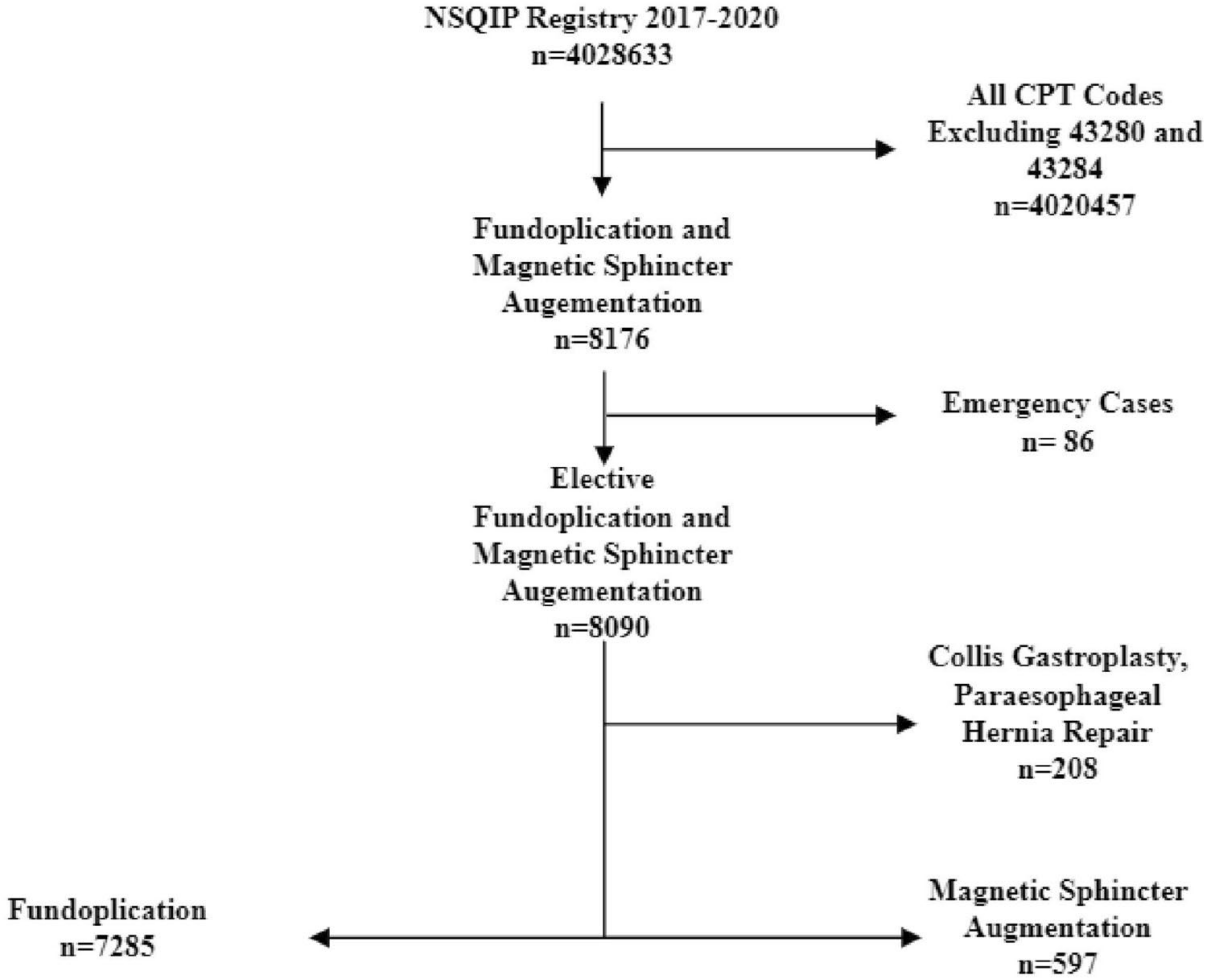
Study flow algorithm

The study population consisted of all patients undergoing MSA and laparoscopic fundoplication between 2017 and 2020 based on Common Procedural Codes 43284 and 43280, respectively. All emergency cases and patients undergoing concomitant paraesophageal hernia repair or Collis gastroplasty were excluded, Fig. 1. The overall complication rate consisted of all postoperative complications including: superficial surgical site infection, deep incisional site infection, organ space infection, wound disruption, pneumonia, unplanned intubation, pulmonary embolism, ventilator over 48 h, progressive renal insufficiency, acute renal failure, urinary tract infection, stroke, cardiac arrest requiring cardiopulmonary resuscitation, bleeding requiring transfusion, deep vein thrombosis, sepsis, or septic shock. Causes of readmission were evaluated using ICD-10 codes which described postoperative complications including gastroesophageal reflux, dysphagia, nausea or vomiting, postoperative pain, ileus or obstruction, seroma or hematoma, gastric motility issues, and diaphragmatic hernia, Supplementary Table 1. Patient demographics, intraoperative details, and 30-day outcome data were reviewed.

### Statistical Analysis

Categorical variables were evaluated with Chi-squared and Fisher’s exact tests. Continuous variables were evaluated using Mann–Whitney *U* for nonparametric data, and Student’s *T* test for parametric data. Significant univariable outcomes on comparative analyses were evaluated using binary logistic regression. Multiple logistic regression with forward stepwise variable selection was used to construct a predictive model. Independent variables with a significant association of *p* < 0.1 were included in the multivariable model. All analyses were performed with IBM Statistical Package for Social Sciences (SPSS).

## Results

### Patient demographics and co-morbid conditions

A total of 7,882 patients were included in the analysis: 7285(92.4%) underwent fundoplication and 597(7.6%) underwent MSA. Patient demographics, perioperative characteristics, and co-morbidities are reported in Table 1. The MSA cohort was younger (51 vs 57 years, *p* < 0.001), with a greater proportion of male patients (49.6 vs 34.3%. *p* < 0.001), lower body mass index (29.5 vs 29.7 kg/m^2^, *p* = 0.018), represented more Caucasian patients (86.4 vs 82.4%, *p* = 0.023), and more Hispanic patients (9.2 vs 5.8%, *p* < 0.002) compared to patients undergoing fundoplication. Additionally, MSA patients presented with fewer medical co-morbidities.

**Table 1:** Demographics and characteristics magnetic sphincter augmentation vs fundoplication

Demographic	Fundoplication (*n* = 7285)	MSA (*n* = 597)	*p*
Age (Median)	57 (45–67)	51 (39–61)	** < 0.001**
Gender			
Female	4785 (65.7%)	301 (50.4%)	** < 0.001**
Male	2500 (34.3%)	296 (49.6%)	
BMI, kg/m^2^	29.7 (26.4–33.4)	29.5 (26.0–32.5)	**0.018**
Race			
Caucasian	6001 (82.4%)	516 (86.4%)	**0.023**
Black or African American	366 (5.0%)	26 (4.4%)	
Other	918 (12.2%)	55 (9.2%)	
Hispanic	420 (5.8%)	55 (9.2%)	**0.002**
Diabetes			
Non-Insulin	499 (6.8%)	33 (5.5%)	0.068
Insulin	206 (2.8%)	9 (1.5%)	
Smoker	863 (11.8%)	49 (8.2%)	**0.008**
Dyspnea			
Moderate	656 (9.0%)	18(3.0%)	** < 0.001**
At Rest	34 (0.5%)	1 (0.2%)	
Ventilator*	5 (0.1%)	0 (0.0%)	1.000
Functional Status*			
Partially Dependent	65 (0.9%)	2 (0.3%)	0.239
Totally Dependent	10 (0.1%)	0 (0.0%)	
COPD	387 (5.3%)	9 (1.5%)	** < 0.001**
CHF*	25 (0.3%)	0 (0.0%)	0.255
Hypertension	2907 (39.9%)	192 (32.2%)	** < 0.001**
Steroid Use	369 (5.1%)	16 (2.7%)	**0.009**
10% Weight loss*	75 (1.0%)	1 (0.2%)	**0.029**
Bleeding disorders*	89 (1.2%)	4 (0.7%)	0.322
ASA classification			
Class 1	153 (2.1%)	17 (2.8%)	** < 0.001**
Class 2	3902 (53.6%)	429 (71.9%)	
Class 3	3091 (42.4%)	147 (24.6%)	
Class 4	129 (1.8%)	3 (0.5%)	

Bold values are statistically significant

*Represents Fisher's Exact Test

*BMI* body mass index, *Diabetes* diabetes mellitus type 2, *COPD* chronic obstructive pulmonary disease, *CHF* congestive heart failure, *ASA classification* American Society of Anesthesiologists classification

### 30-day operative and post-operative outcomes

Patients undergoing MSA had shorter operative time (80.8 vs 118.7 min, *p* < 0.001) and hospital length of stay (0.4 vs 1.8 days, *p* < 0.001), Table 2. MSA patients had lower rates of postoperative pneumonia (0.2 vs 1.0%, *p* = 0.044) and urinary tract infections (0.0 vs 0.9%, *p* = 0.009). There was no significant difference in cerebrovascular cardiac, bleeding, or thromboembolic complications between the two groups. MSA patients had lower rates of readmissions (2.2 vs 4.7%, *p* = 0.005), and overall complications (0.6 vs 4.0%, *p* < 0.001). The causes for readmissions between groups were similar (Table 3). There were no significant differences in mortality (0.00 vs 0.03%, *p* = 0.396).

**Table 2:** Perioperative outcomes magnetic sphincter augmentation vs fundoplication

Outcomes	Fundoplication (*n* = 7285)	MSA (*n* = 597)	*p*
Operative Time (min)	118.7 ± 63.7	80.8 ± 36.1	** < 0.001**
Length of Stay (days)	1.8 ± 4.6	0.4 ± 4.3	** < 0.001**
Superficial skin infection	29 (0.4%)	1 (0.2%)	0.725
Deep incisional infection	2 (0.0%)	0 (0.0%)	1.000
Organ space infection	40 (0.5%)	0 (0.0%)	0.072
Wound disruption	5 (0.1%)	0 (0.0%)	1.000
Pneumonia	74 (1.0%)	1 (0.2%)	**0.044**
Unplanned intubation	28 (0.4%)	0 (0.0%)	0.268
Pulmonary embolism	22 (0.3%)	1 (0.2%)	1.000
Ventilator > 48 h	18 (0.2%)	0 (0.0%)	0.393
Renal insufficiency	5 (0.1%)	1 (0.2%)	0.377
Acute renal failure	6 (0.1%)	0 (0.0%)	1.000
Urinary tract infection	67 (0.9%)	0 (0.0%)	**0.009**
CVA	3 (0.0%)	0 (0.0%)	1.000
Cardiac arrest	10 (0.1%)	0 (0.0%)	1.000
Myocardial infarction	26 (0.4%)	0 (0.0%)	0.258
Bleeding	33 (0.5%)	0 (0.0%)	0.174
DVT	18 (0.3%)	0 (0.0%)	0.393
Sepsis	21 (0.3%)	0 (0.0%)	0.400
Septic shock	20 (0.3%)	0 (0.0%)	0.396
Reoperation	144 (2.0%)	6 (1.0%)	0.095*
Readmission	339 (4.7%)	13 (2.2%)	0.005*
Overall complications	395 (4.0%)	4 (0.6%)	** < 0.001**
Mortality	20 (0.03%)	0 (0.0%)	0.396

Bold values are statistically significant

^*^Represents Chi-squared analysis

*CVA* cerebrovascular accident, *DVT* deep vein thrombosis

**Table 3:** Causes of readmission magnetic sphincter augmentation vs fundoplication

Causes	Fundoplication (*n* = 7285)	MSA (*n* = 597)	*p*
GERD	17 (0.2%)	0 (0.0%)	0.634
Dysphagia	34 (0.5%)	3 (0.5%)	1.000
Nausea/vomiting	20 (0.3%)	2 (0.3%)	0.681
Postoperative pain	28 (0.4%)	0 (0.0%)	0.268
Ileus/obstruction	18 (0.3%)	0 (0.0%)	0.393
Seroma/hematoma	2 (0.0%)	0 (0.0%)	1.000
Cellulitis	1 (0.0%)	0 (0.0%)	1.000
Gastric dysmotility	5 (0.1%)	0 (0.0%)	1.000
Diaphragmatic hernia	5 (0.1%)	0 (0.0%)	1.000

*GERD* gastroesophageal reflux disease

### Multivariable regression outcomes

On multivariable logistic regression, MSA patients had fewer cases of pneumonia and urinary tract infections although these outcomes did not reach statistical significance. However, MSA patients did have a significantly lower rate of readmission (OR 0.534, *p* = 0.029) and overall complications (OR 0.228, *p* = 0.004), Table 4. Multivariable linear regression demonstrated that MSA was associated with significantly reduced length of hospitalization (RC = − 1.222, *p* < 0.001), and reduced operative time (RC = − 36.558, *p* < 0.001), Table 5.

**Table 4:** Multivariable regression examining the association of postoperative complications of Magnetic Sphincter Augmentation (MSA) vs Fundoplication (Fundoplication is baseline)

	Univariable				Multivariable			
	95% C.I.				95% C.I.			
	OR	Lower	Upper	*p*	OR	Lower	Upper	*p*
Pneumonia	0.163	0.023	1.178	0.072	–	–	–	–
Urinary tract infection	0.000	0.000	–	0.995	–	–	–	–
Readmission	0.456	0.260	0.799	0.006	0.534	0.303	0.939	0.029
Overall complications	0.160	0.059	0.430	< 0.001	0.228	0.084	0.616	0.004

Covariates used in creation of multivariable model: Gender, Race, Ethnicity, Operation Type, Age, Body Mass Index, Diabetic Status, Smoking History, Dialysis Requirement, Functional Status, Dyspnea, COPD, Congestive Heart Failure, Hypertension, Steroid Use, Bleeding Disorders, > 10% weight loss over 6 months, and American Society of Anesthesiologists Classification System

**Table 5:** Multivariable linear regression of continuous outcomes (Fundoplication is baseline)

Outcome	Unstandardized regression coefficient	95% CI	*p*
Length of stay (days)	− 1.222	− 1.606 to − 0.837	< 0.001
Operative time (min)	− 36.558	− 41.623 to − 31.493	< 0.001

Covariates: Age, Sex, Preoperative Body Mass Index, American Society of Anesthesiologists Classification, Operation type, Smoking and Clinical History: (Functional Status, Hypertension, Diabetes, Chronic Obstructive Pulmonary Disease, and Congestive Heart Failure)

## Discussion

Gastroesophageal reflux disease is a common disease that affects at least one out of every five Americans. Despite medical optimization, nearly half of all patients experience persistent GERD symptoms [1, 4]. Traditional surgical therapy involves a fundoplication, which can have frequent and bothersome side effects and complications. Alternatively, magnetic sphincter augmentation is a newer anti-reflux operation with a potentially reduced complication rate.

The only contraindication to the use of MSA is metal allergy. The Food and Drug Administration additionally lists multiple precautionary conditions such as Age < 21, body mass index (BMI) > 35, pregnancy status, and various esophageal functional, and structural comorbidities [6]. In the author’s institution, we maintain avoiding device implantation in patients under 21 years of age, pregnancy, malignancy, esophageal varices, and those with concurrent electrical implants. Aside from these is used in broadly including in patients with precautionary conditions with a preoperative discussion reviewing the risks of failure and need for reintervention or explantation. Several studies have demonstrated the safety of MSA in esophageal dysmotility, advanced esophagitis, Barrett’s esophagus, and BMI > 35 [7–9]. In this study, despite differing baseline demographics and co-morbidities, there were few postoperative differences noted between groups on comparative analysis. MSA patients experienced reduced operative time and length of stay which is consistent with other contemporary studies. In a prospective, multi-institutional study, Reynolds et al. reported a mean MSA operative time of 60 min and mean length of stay of 11 h [10]. Other studies have demonstrated similar operative times in a range 40–66 min in patients undergoing MSA, compared 76–90 min in patients undergoing Nissen fundoplication [11–14]. Furthermore, while early trials on MSA monitored patients overnight, same day discharge has been shown to be safe [13, 14] Lastly, a recent meta-analysis showed MSA was associated with reduced operative time and length of stay compared to fundoplication [15]. These findings likely result from the elimination of significant esophago-gastric mobilization and fixation required during fundoplication.

Rates of postoperative complications were slightly different between the MSA and fundoplication patients Those undergoing fundoplication experienced greater rates of urinary tract infections, pneumonia, readmissions, and overall complications. In a propensity matched study of 100 patients undergoing MSA vs Nissen fundoplication, Reynolds et al. demonstrated no complications at 1 year, while 2 major complications (dehydration and esophageal obstruction) and 2 minor complications (postoperative seizure and urinary tract infection) were noted in the Nissen cohort [13]. In a study examining the first 1000 MSA cases utilizing the Manufacturer and User Facility Device Experience Database, Lipham et al. reported readmission rates of 1.3% over a 90-day postoperative period. 0.8. When comparing similar readmission criteria, the overall rate of readmission for this study is 0.8%; however, this is not an equal comparison as the NSQIP database captures 30-day complications compared to 90-day complication in the Manufacturer’s database [16].

Prolonged operative time has been shown to be associated with increased perioperative complications. In an ACS- NSQIP analysis, Jackson et al. showed patients undergoing minimally invasive Nissen fundoplication demonstrated increasing odds of complications with increasing operative time (*p* < 0.001) [17]. Similarly in a recent systematic review, Chen et al. showed increasing complications with increasing operative time across multiple surgical specialties [15]. In our study, operative time was significantly longer in patients undergoing laparoscopic fundoplication and this may, in part, explain the findings of increased overall complication rates seen in this study.

The limitations of this study are inherent to the ACS-NSQIP database. First, data are available for only the first 30 days postoperatively. While this is valuable in the assessment of safety, studies have demonstrated the development of long-term complications such as dysphagia, and MSA device erosion requiring intervention or explantation. Second, the database is unable to distinguish between partial and Nissen fundoplication. MSA is more comparable to a Nissen fundoplication given the circumferential positioning of the device. Additionally, different fundoplication techniques such as anterior or posterior wraps, or retention of the short gastric vessels, may skew outcome data. Finally, objective and subjective data on GERD outcomes is lacking such as esophagram, esophageal pH studies, histopathological data, and standardized patient GERD quality of life questionaries which would allow for an examination of efficacy outcomes of MSA vs fundoplication.

## Conclusion

This is the first known large database evaluation of the short-term outcomes of MSA compared to minimally invasive fundoplication. Most studies on MSA up to now utilize data from high-volume institutions; in contrast, NSQIP represents cases from more than 600 hospitals allowing for more generalizable data. This study demonstrates reduced operative time, hospital stay, readmission rates, and overall complications for patients undergoing MSA compared to fundoplication. While these data represent 30-day outcomes, MSA is a safe alternative to minimally invasive fundoplication.

### Supplementary Information

Below is the link to the electronic supplementary material.

Supplementary file1 (DOCX 13 KB)

## Declarations

### Disclaimers

The American College of Surgeons National Surgical Quality Improvement Program and the hospitals participating in the ACS NSQIP are the source of the data used herein; they have not verified and are not responsible for the statistical validity of the data analysis or the conclusions derived by the authors**.**
