# Supplementary material for: Short term safety of magnetic sphincter augmentation vs minimally invasive fundoplication: an ACS-NSQIP analysis
Source: Surg Endosc. 2024 Feb 9;38(4):1944–9. doi: 10.1007/s00464-024-10672-7 (PMC10978616; doi:10.1007/s00464-024-10672-7)
Supplement: Supplementary file 1 — Supplementary file1 (DOCX 13 KB) [file 464_2024_10672_MOESM1_ESM.docx]

| **Supplementary Table 1. Causes of Readmission for MSA vs Fundoplication** | |
| --- | --- |
| Readmission Reasons | ICD-10 Codes |
| GERD | K20.8, K21.0, K21.9, K22.10, K22.6, K22.8, K25.9, K27.4, K29.0, K29.01, K29.60, K30 |
| Dysphagia | I69.891, K22.2, R13.1, R13.10, R13.11, R13.14, R13.19, R47.01, |
| Nausea/vomiting | R11, R11.0, R11.10, R11.2, K91.0 |
| Postoperative Pain | G89.18, R10.11, R10.12, R10.13, R10.8, R10.84, R10.9, R07.0, R07.89, R07.9 |
| Ileus/Obstruction | K56.5, K56.60, K56.69, K56.7, K59.0, K59.00, K91.30 |
| Seroma/Hematoma | K91.872 |
| Cellulitis | L03.90 |
| Gastric motility | K31.0, K31.1, K31.84 |
| Diaphragmatic hernia | K44.0, K 44.9 |
